# Hematologic Findings in Pregnancy: A Guide for the Internist

**DOI:** 10.7759/cureus.15149

**Published:** 2021-05-21

**Authors:** Pooja Patel, Nino Balanchivadze

**Affiliations:** 1 Internal Medicine, Grand Strand Medical Center, Myrtle Beach, USA; 2 Hematology and Oncology, Henry Ford Health System, Detroit, USA

**Keywords:** pregnancy, hematologic manifestations, anemia in pregnancy, thrombocytopenia in pregnancy, bleeding in pregnancy, thromboembolism in pregnancy

## Abstract

Hematologic changes in pregnancy are common and can potentially lead to maternal and fetal morbidity. Here, we present various hematologic manifestations seen in pregnant women. Iron deficiency anemia (IDA) is the most common cause of anemia in pregnancy. Physiologically, the state of pregnancy results in increased iron demand. Iron deficiency is important to diagnose and treat early for better maternal and fetal outcomes. An algorithmic approach is used for the repletion of iron storage, starting with oral elemental iron daily and escalating to intravenous iron if necessary. Folate and cobalamin are necessary elements for deoxyribonucleic acid (DNA) synthesis, fetal growth, and maternal tissue development, and deficiency in these elements can be a cause for anemia in pregnancy. Thrombocytopenia is currently the second most common hematologic condition in pregnancy after anemia. There is a wide range of etiology for thrombocytopenia in pregnancy from benign to life-threatening causes that require prompt diagnosis and treatment. These conditions include gestational thrombocytopenia, thrombotic thrombocytopenic purpura, pregnancy-associated atypical hemolytic-uremic syndrome, and immune thrombocytopenia. Acquired bleeding disorders that can cause major complications in pregnancy include von Willebrand disease (vWD) and coagulation factor deficiencies. Women with vWD are at increased risk of pregnancy bleeding and postpartum hemorrhage. Pregnancy can also produce a physiologic hypercoagulable state, leading to life-threatening conditions like thromboembolism. Diagnosis, treatment options, and guidelines for the management of these conditions will be explored in this review.

## Introduction and background

Pregnancy induces a number of direct and indirect physiological changes in women, and various hematologic manifestations have been described. Anemia is a common and significant maternal problem, and anemia during pregnancy most commonly results from a nutritional deficiency in iron or folate [1]. Another hematological disorder, thrombocytopenia, is also a common complication in pregnancy [2]. Differentiating gestational thrombocytopenia from immune thrombocytopenia (ITP), pregnancy-associated atypical hemolytic-uremic syndrome (p-aHUS), and thrombotic thrombocytopenic purpura (TTP) is essential because effective treatments for these conditions vary [3]. Congenital and acquired bleeding disorders, such as von Willebrand disease (VWD), and acquired coagulation factor deficiencies can present with hemostatic challenges during various stages of pregnancy, with increased risk of bleeding and mortality [4]. Pregnant patients encounter hematological conditions frequently, thus it is important as an internist to understand the pathophysiology and presentation of most commonly occurring disease processes. This article will review the diagnosis and management of iron deficiency anemia, folate and cobalamin deficiency, thrombocytopenia, atypical HUS, acquired bleeding disorders, venous thromboembolism, coagulation abnormalities, and the role of novel anticoagulants in pregnancy. Prompt diagnosis and treatment of these hematologic conditions is imperative to avoid poor outcomes in pregnant patients and developing fetuses.

## Review

Anemia

Iron Deficiency

The Centers for Disease Control and Prevention (CDC) defines anemia as a hemoglobin concentration of <11 g/dL (hematocrit of <33%) within the first or third trimester or a hemoglobin concentration of <10.5 g/dL (hematocrit <32%) in the second trimester of pregnancy [1]. Iron deficiency anemia (IDA) is the most common pathologic cause of anemia in pregnancy since pregnancy results in increased iron demand [5]. Iron requirements during pregnancy vary between 800 and 1000 mg, depending on the size of the woman (45-55 kg), with most of the extra requirements occurring in the second half of pregnancy [6]. The demand for absorbed iron increases from approximately 0.8 mg/day in early pregnancy to 7.5 mg/day in late pregnancy [7].

Symptoms of IDA are caused by decreased oxygen delivery to the tissues and include pallor, fatigue, apathy, fainting, and breathlessness. Nutritional iron intake is the key to preventing iron deficiency, and the total iron intake during the course of a pregnancy should not be less than 1000 mg [8]. Iron deficiency during the first trimester has a more negative impact on fetal growth than anemia that develops later in pregnancy [9]. There is also an increased risk of developing a perinatal infection, preeclampsia, and bleeding associated with IDA [10].

Diagnosis of IDA in pregnant women can be challenging because the changes in maternal physiology may affect the serum levels of biochemical markers of iron status. Serum ferritin, the storage form of iron, is the most specific initial test for diagnosing iron deficiency in pregnant women [11]. Serum ferritin concentration can be elevated in various conditions of inflammation, and using other markers to support the diagnosis of IDA may be warranted. A decreased transferrin saturation or an elevated serum soluble transferrin receptor concentration in a pregnant patient who has a normal or elevated serum ferritin concentration could be useful in confirming IDA [12].

Achebe and colleagues have suggested an algorithm for diagnosing and managing IDA [13]. A serum ferritin concentration < 30 μg/L together with a hemoglobin concentration <11 g/dL during the first trimester, <10.5 g/dL during the second trimester, and <11 g/dL during the third trimester are all diagnostic for anemia during pregnancy [14].

The first-line treatment for IDA in pregnancy is oral iron, with suggested replacement of 40 to 200 mg of oral elemental iron daily. Reassessing hemoglobin levels within two weeks will evaluate the response and confirm adequate absorption when there is an increase of hemoglobin by 1 gram. Oral iron supplementation should continue for two to three months after normalization of hemoglobin and then six weeks postpartum [13]. If oral iron is not tolerated because of side effects (such as constipation or heartburn) or if there is insufficient absorption, intravenous iron can safely be given. Intravenous iron corrects hemoglobin and iron stores concomitantly [12]. A meta-analysis of 103 randomized controlled trials that compared 10,391 patients who received intravenous iron, 4044 patients who received oral iron, 1329 who received no iron, and 3335 who received placebo showed that intravenous iron therapy did not lead to serious adverse events or increased rate of infections [15]. Iron sucrose and sodium ferric gluconate are assigned as Food and Drug Administration (FDA) pregnancy category B therapies based on safety studies in pregnant women [13]. However, both high molecular weight and low molecular weight (LMW) iron dextrans retain a pregnancy category C designation, despite evidence suggesting that adverse events ascribed to iron dextran are mostly associated with the high molecular weight formulation [16]. Some investigators have reported additional advantages of intravenous over oral iron therapy beyond the more rapid increase in hemoglobin. Breymann and colleagues enrolled 252 women who were in their second or third trimester (weeks 16-33) and randomly assigned them to oral ferrous sulfate or intravenous ferric carboxymaltose [17]. Hemoglobin level improvements and newborn outcomes were similar in both groups, but vitality and social functioning were better in the group that received intravenous iron [17]. All intravenous formulations may be associated with allergic reactions characterized by nausea, hypotension, tachycardia, chest pain, dyspnea, and edema of the extremities, and they mostly occur within 24 hours of the infusion. These minor infusion reactions are self-limited and do not require treatment [18]. In a prospective study, Froessler and colleagues treated 65 pregnant women with fibrin monomer complex (FCM) and reported no serious adverse effects and no change in fetal heart monitoring [19]. Furthermore, Christoph et al. evaluated 206 pregnant women in a comparison study of iron sucrose and FCM and showed equivalent safety profiles for both drugs [20].

Folate and Cobalamin Deficiency

Folate deficiency was the second most common cause of anemia before nationwide mandatory folate fortification programs were initiated [21]. Folate deficiencies vary among pregnant women, from 1% to 50%, and is higher in low socioeconomic regions of the world. Folate and vitamin B12 (cobalamin) are involved in tetrahydrofolate metabolism and are necessary for deoxyribonucleic acid (DNA) synthesis, fetal growth, and maternal tissue development [22]. Dietary folate is absorbed in the jejunum, and inadequate nutrition, intestinal malabsorption, and increased requirements for fetal growth contribute to folate deficiency. Cobalamin plays a critical role in DNA synthesis, and hematopoietic precursor cells are highly sensitive to abnormal DNA synthesis caused by cobalamin deficiency. While mild anemia, leukopenia, and thrombocytopenia are common, 10% of patients with cobalamin deficiency develop life-threatening hematologic manifestations such as hemolytic anemia [23]. Cobalamin is present in animal protein and absorbed in the terminal ileum. R-protein (haptocorrin), secreted by salivary glands, binds cobalamin in the stomach and transports cobalamin to the duodenum where pancreatic proteases degrade the R-protein. Cobalamin is then released and binds to an intrinsic factor released from gastric parietal cells. The cobalamin intrinsic factor complex subsequently binds to receptors on ileal enterocytes. Atrophic gastritis, proton pump inhibitors, and malabsorption all increase the risk of cobalamin deficiency [24].

Diagnostic testing for folate and cobalamin deficiency has some challenges. Folate and cobalamin deficiency can be masked by iron deficiency and do not always result in macrocytosis [24]. Given that the biochemical pathways of cobalamin and folate are closely intertwined, with patients showing similar clinical features for both deficiencies, assessment of cobalamin and folate status is usually performed concurrently [25]. Serum folate is seldom normal and is often elevated within the context of true cobalamin deficiency. However, a low serum cobalamin level can also indicate the presence of folate deficiency. A serum cobalamin assay is currently the standard initial routine diagnostic test [26], and cobalamin deficiency results in elevated plasma total homocysteine. However, total homocysteine is not specific to cobalamin deficiency, as concentrations of homocysteine are also elevated during folate deficiency and B6 deficiency, and in patients with renal failure or hypothyroidism, and it may be the result of certain genetic polymorphisms [27]. Plasma methylmalonic acid is raised in cobalamin deficiency; however, it may also be falsely elevated in patients with renal disease, small bowel bacterial overgrowth, or hemoconcentration. Despite these limitations, exceptionally high levels of plasma methylmalonic acid (> 0·75 μmol/L) almost invariably indicate cobalamin deficiency. A serum cobalamin cutoff level of either 148 pmol/L (200 ng/L) or one derived from a local reference range should be used as evidence of cobalamin deficiency and should be considered as strong evidence of a true deficiency. The interpretation of the result should be considered in relation to clinical circumstances [28]. As for folate, serum folic acid concentrations < 2 ng/mL are diagnostic of folic acid deficiency, whereas levels above 4 ng/mL effectively rule out deficiency, and levels in the range of 2 to 4 ng/mL are borderline [29].

The World Health Organization (WHO) recommends folate supplementation of 400 μg per day for pregnant women from early pregnancy to three months post-partum. The US Public Health Service and CDC recommend the same for all women of childbearing age (15-45 years of age) to prevent spina bifida and anencephaly [30]. Most prenatal vitamins contain 1 mg of folate, which is more than sufficient to meet the increased needs of pregnancy [13]. A higher supplementation dosage, such a 5 mg per day, is recommended for women who have increased demands for folate (multiple pregnancies, hemolytic disorders, folate metabolism disorders) and for women who are at an increased risk of neural tube defects (personal or family history of neural tube defect, pre-gestational diabetes, or epilepsy on valproate or carbamazepine) [31]. Regarding cobalamin supplementation recommendations, the WHO and US National Institutes of Health recommend a higher daily allowance of cobalamin in pregnant women than in nonpregnant women (2.6 vs 2.4 μg per day) [32].

Thrombocytopenia

Thrombocytopenia is defined as a platelet count <150 × 109/L. It is the second most common hematologic condition in pregnancy after anemia and is usually a benign condition [33]. Approximately one in 10 pregnant women will develop thrombocytopenia during an otherwise unremarkable pregnancy [34]. The American Society of Hematology recommends that treatment should be initiated for platelet counts below 30,000/mm^3^ or in cases of bleeding within the second or third trimesters of pregnancy [34-36]. Etiologies for thrombocytopenia in pregnancy range from benign (eg, gestational thrombocytopenia) to life-threatening (eg, hemolysis) and may present as HELLP (hemolysis, elevated liver enzymes, and low platelets) syndrome, TTP, or ITP [37].

Gestational thrombocytopenia accounts for 70% to 80% of cases [38]. It is usually an incidental diagnosis, and its pathogenesis remains unclear. Two mechanisms have been thought to play a role in gestational thrombocytopenia, including hemodilution and accelerated platelet clearance [39]. In a prospective study, 22 pregnant women with incidental thrombocytopenia (<100,000/μl) were monitored for platelet count and clinical outcome for a minimum of six months postpartum [40]. The thrombocytopenia was virtually asymptomatic in all patients during the pregnancy and delivery, whether vaginal or surgical. It was concluded that gestational thrombocytopenia of <100,000/μl is clinically a benign phenomenon that can recur in subsequent pregnancies and is not accompanied by neonatal thrombocytopenia. In some cases, however, pregnancy‐associated thrombocytopenia may be a manifestation of an autoimmune disease with attendant implications for the neonate. Since the differential diagnosis between the two conditions may be difficult to establish when first encountered during pregnancy, a conservative approach emphasizing careful surveillance and guarded reassurance is justified, as long as the platelet counts are >50,000/μl [40].

ITP is the second-most common cause of an isolated low platelet count in pregnancy, accounting for ∼3% of women who are thrombocytopenic at delivery. As a general rule, a platelet count < 100 × 109/L in early pregnancy accompanied by declining platelet counts as gestation progresses is most consistent with ITP [4]. A careful review of the peripheral blood smear remains the main diagnostic procedure. ITP should be suspected when an otherwise healthy pregnant woman (bleeding excepted) who has no relevant family or gestational history of anemia and who is not taking medications that would increase the risk of ITP presents with a platelet count below 70 × 109/L to 80 × 109/L in the first or second trimester and has a peripheral blood smear notable only for thrombocytopenia without unusually small or giant platelets. The treatment of chronic ITP aims to maintain the platelet count in a state that does not cause bleeding and other complications (above 50,000/mm^3^) [41]. The first-line drugs usually recommended for treating ITP include corticosteroid and intravenous immunoglobulin (IVIG) therapy [42]. When a patient does not respond to corticosteroids and IVIG therapy, splenectomy is indicated as the second option [43-45]. A study carried out in 2014 demonstrated the higher incidence of premature birth and postpartum infection in women who needed corticosteroid therapy in pregnancy than in those who had not been treated with the drug. In addition, when corticosteroids are used in the first trimester of pregnancy, congenital anomalies such as orofacial clefts may occur as a consequence [36]. The American Society of Hematology guidelines state that IVIG is an appropriate first-line agent for severe thrombocytopenia or bleeding due to thrombocytopenia in the third trimester of pregnancy [43]. Generally, treating ITP during pregnancy shows successful results. However, more studies assessing maternal and fetal outcomes after the postpartum period are needed to confirm the long-term outcomes after treatment.

TTP during pregnancy can be either a de novo manifestation of disease or a recurrence of a previously known TTP triggered by the pregnant state [46]. Congenital and acquired deficiency of the von Willebrand (vWD) factor cleaving protease (disintegrin and metalloprotease with thrombospondin-1-like domains; ADAMTS13) are considered the hallmark of TTP pathophysiology [47]. The majority of acute cases are acquired, autoantibody-mediated, and are characterized by the presence of anti-ADAMTS13 immunoglobulin G (IgG) antibodies and low ADAMTS13 activity (<10%). Acquired TTP is postulated to be secondary to viral infections that trigger the production of autoantibodies targeted against ADAMTS13 [48]. A small proportion of TTP cases are attributable to congenital disease, with low ADAMTS13 activity (<10%) and no detectable antibody, confirmed by mutational analyses. The pentad of clinical features associated with TTP includes thrombocytopenia, microangiopathic hemolytic anemia, fever, and neurological and renal abnormalities [49]. There is some evidence of TTP presenting in the second trimester; however, a British prospective study described the majority of presentations as having occurred after 30 weeks gestation [49-50]. This demonstrates that TTP can present during different gestational stages.

Treatment options for TTP include cryosupernatant plasma-based plasmapheresis and platelet transfusion, and these therapies have favorable maternal and neonatal outcomes. Cryosupernatant plasma is a viable alternative to fresh frozen plasma for plasmapheresis treatment of TTP and may offer some therapeutic and logistical advantages [51]. In either case, the mainstay treatment for TTP remains plasma exchange.

A rare condition characterized by microangiopathic hemolytic anemia, thrombocytopenia, and acute kidney injury is p-aHUS [52]. This syndrome is triggered by pregnancy, in which genetically predisposed women develop hemolytic disease characterized by diffuse endothelial damage and platelet consumption. It affects one out of every 25,000 pregnancies, mostly in the postpartum period, and it is associated with poor maternal outcomes [53].

The diagnosis of p-aHUS can be difficult, as this condition is non-specific in its symptom presentation and can mimic other diseases that must be ruled out when making a diagnosis. The common clinical presentation of p-aHUS is acute renal failure, thrombocytopenia, and microangiopathic hemolytic anemia [54], however, these features are also observed in TTP, acute fatty liver of pregnancy, and severe preeclampsia with HELLP syndrome. Although distinguishing between these syndromes is sometimes difficult, making the right diagnosis in a timely manner is imperative. HELLP syndrome typically resolves after delivery, and hemolysis becomes less severe [55]. However, HELLP syndrome stimulates the development of p-aHUS in genetically predisposed patients [52].

Atypical HUS is a life-threatening condition that requires prompt diagnosis and therapy, whether it is within the context of pregnancy or not. Initial management includes immediate plasma exchange therapy while the diagnostic workup is underway. High-dose weight-based steroids (prednisone 1 mg/kg/day) can also be used. Renal replacement therapy should also be used for hyperkalemia, pulmonary edema, metabolic acidosis, and uremia [52]. The pathogenesis of atypical HUS is overactivation of the complement pathway. Eculizumab is a humanized monoclonal antibody that halts the complement cascade by binding to C5 and blocking its conversion to the active complement form of membrane attack complex [56]. Women with p-aHUS have shown improved platelet function and renal function from eculizumab therapy, which decreases the need for dialysis. This advancement has improved the quality of life in patients with atypical HUS [57].

Acquired bleeding disorders

The process of hemostasis is complex and is further complicated in the state of pregnancy, and women with inherited bleeding disorders face hemostatic challenges during various physiological stages of pregnancy. Acquired bleeding disorders that can cause major issues include vWD factor and coagulation factor deficiencies [58]. The most common inherited bleeding disorder in the general population is vWD, affecting up to 1% of the general population [59]. The vWF is a large, multimeric protein with two crucial roles in hemostasis: it serves as a bridging molecule between subendothelial collagen exposed during vascular injury and platelets and it acts as a chaperone protein for factor VIII, preventing premature degradation and increasing availability at sites of active thrombus formation [60]. During pregnancy, hormonal fluctuations lead to an increase in vWF and clotting factors VII, VIII, and X while anticoagulant factors (such as protein S) decrease, shifting hemostasis to a procoagulant state to compensate for anticipated hemorrhage during parturition [61].

Women with vWD are at increased risk of pregnancy bleeding and postpartum hemorrhage, which are pregnancy complications due to the loss of a protective prothrombotic state [62]. For patients without a previous diagnosis, a history of excessive menstrual or mucocutaneous bleeding, previous postpartum hemorrhage, bleeding after surgical/dental procedures, or a family history of bleeding prompts diagnostic testing. Diagnosis starts with complete blood count to evaluate platelets and standard coagulation studies (ie, prothrombin time and partial thromboplastin time) [63]. Recommended screening tests include plasma vWF antigen, vWF activity (with ristocetin cofactor activity being most commonly performed and the collagen-binding assay less readily available), and factor VIII activity [64]. Abnormal vWF levels (<30%-40%) and/or a low vWF ristocetin cofactor to vWF antigen ratio should prompt additional testing such as vWF multimer pattern using gel electrophoresis and ristocetin-induced platelet aggregation. Confirmatory molecular analysis should be performed to verify the diagnosis [65].

Treatment options for vWD include desmopressin (1-8-deamino-D-arginine vasopressin, DDAVP), a synthetic analog of vasopressin, to increase plasma factor VIII and vWF levels transiently in patients with vWD and in non-affected individuals with a bleeding disorder. The safety and efficacy of DDAVP for prophylaxis or treatment of pregnancy-associated bleeding have not been systematically studied [61]. DDAVP may not be effective for type 2 vWD and is contraindicated for type 3 vWD, where its use may lead to worsening thrombocytopenia. Patients with type 3 vWD generally do not respond to DDAVP. When DDAVP cannot be given, replacement therapy with plasma-derived vWF and factor VIII concentrates or recombinant vWF should be initiated [63]. Antifibrinolytic tranexamic acid has gained attention as an anti-bleeding agent. In an international, randomized, double-blinded, placebo-controlled trial named WOMAN (world maternal antifibrinolytic trial), tranexamic acid reduced deaths due to bleeding with no observed increase in thromboembolic events. The effect was greatest when women received tranexamic acid within three hours of childbirth (relative risk, 0.69; 95% CI, 0.52-0.91) [66]. WHO recommendations have recently changed to recommend that women with postpartum hemorrhage should receive 1 g of tranexamic acid intravenously as soon as possible after giving birth, followed by a second dose if bleeding continues after 30 min or restarts within 24 hours of the first dose [67]. This recommendation applies to women with vWD as well.

Venous thromboembolism and coagulation abnormalities

Pregnancy produces a physiological hypercoagulable state that is caused by changes in the coagulation system [68]. As pregnancy progresses, the risk of hypercoagulability increases because of decreasing levels of protein S and increasing levels of thrombogenic factors VII, VIII, and X; vWF; and fibrinogen [69]. The risk of coagulation complications, such as venous thromboembolism, is highest in the immediate postpartum period, and the risk slowly decreases back to pre-pregnancy levels by eight to 12 weeks post-partum [70]. Thrombolysis is a common therapeutic option for nonpregnant women with thrombosis. However, very few randomized controlled trials of thrombolytic therapy have included pregnant women; therefore, the risks and benefits of thrombolysis in this population are unknown. Recent studies have suggested a link between thrombophilia and adverse pregnancy outcomes such as fetal loss and venous thromboembolism [71]. The use of anticoagulant therapy during pregnancy is challenging because of the potential for fetal and maternal complications; therefore, the risks versus the benefits of anticoagulation therapy for pregnant women in the thrombophilic state must be carefully assessed, as it is associated with increased maternal morbidity and mortality [72].

The preferred choice of anticoagulation therapy during pregnancy is LMW heparin with adjusted-dose unfractionated heparin. LMW heparin is the preferred choice for anticoagulation during pregnancy because it has well-demonstrated efficacy and safety in pregnant women [73]. Vitamin K antagonists are not an option in pregnancy, as it is well known for causing fetal embryopathy [74]. LMW heparin is considered superior to unfractionated heparin because it incurs a lower risk of heparin-induced thrombocytopenia. It is recommended to administer unfractionated heparin to reduce hemorrhagic complications cautiously, such as 36 hours prior to the induction of labor or planned cesarean delivery [75].

Adverse reactions to LMW heparin include urticarial rash (type I hypersensitivity reaction), skin necrosis due to vasculitis (type III reaction), and heparin-induced thrombocytopenia, although these risks are lower than those attributed to unfractionated heparin [76]. Fondaparinux is the preferred anticoagulation therapy for patients with heparin-induced thrombocytopenia or anaphylactic allergy to enoxaparin [77]. A retrospective study that assessed women with a history of recurrent (≥ 3) miscarriage and/or a history of infertility with acquired or hereditary thrombophilia showed that fondaparinux was well-tolerated, and no increase in birth defects, severe bleeding-related complications, or serious allergic reactions were observed relative to enoxaparin therapy [78]. Fondaparinux should be used with caution because it can cross the transplacental passage, potentially resulting in measurable anti-factor Xa in umbilical cord blood. It is a matter of concern that in a population-based registry study, one out of 65 pregnancies treated with fondaparinux was complicated by multiple congenital anomalies, a rate of 1.5% (95% CI, 0.3%-8.2%) [79]. Little is known about the use of fondaparinux as a first-line agent for anticoagulation therapy in pregnant women; thus, its use should be limited to cases of documented allergy or adverse response to LMW heparin.

New oral anticoagulant agents (NOACs) are small molecules that have been shown to cross the placenta, and the clinical risk of embryopathy with NOAC use is unknown [80]. A retrospective study that looked at 169 NOAC exposures in pregnancy has been done [81]. The NOACs in the study included rivaroxaban (n = 143), dabigatran (n = 25), and apixaban (n = 1), and venous thromboembolism was the indication for NOAC use in > 95% of the cases. The duration of NOAC exposure ranged from a single dose to complete use through the 2nd and 3rd trimesters. Information on pregnancy outcomes was available in 85 of 169 cases (50.3%) and consisted of 31 live births (36.5%), 21 miscarriages/abortions (24.7%), and 27 elective pregnancy interruptions (31.8%) while six pregnancies (7.1%) were still ongoing at the time of the assessment. Of the 20 live births, three newborns had abnormalities, which included renal pelvis dilatation and facial dysmorphism in one (rivaroxaban exposure in 1st trimester) and low birth weight in two newborns (girl of 2570 g born in week 37 with dabigatran exposure in the 1st trimester and girl of 1175 g delivered by cesarean section for preeclampsia in week 30 with rivaroxaban exposure in the 1st trimester). Of the 21 miscarriages, details were available for five cases (4 miscarriages occurred in the 1st trimester and 1 occurred in week 20 with preceding growth restriction). Of the 27 elective pregnancy interruptions, details were available for seven cases and decisions were based on social reasons (n = 3), fear of NOAC embryopathy (n = 3), and medical reasons (maternal thyrotoxicosis with heart failure) [81]. Further initiatives are needed to more thoroughly investigate the risk of embryopathy from NOAC exposure. A study by Beyer-Westendorf et al. showed that the quality of data on the effects of NOAC exposure in pregnancy that are provided on request from manufacturers and drug authorities is inferior to the quality of data obtained directly from physicians, which indicates a need to improve exposure and outcome assessment in this important medical scenario [81].

## Conclusions

Pregnancy can be associated with myriad hematologic manifestations. Prompt diagnosis and early treatment are often warranted to avoid maternal and fetal complications. General internists should have a thorough knowledge of the hematological complications that are most likely to arise in pregnant women so that prompt diagnoses can be made and effective therapies can be initiated.
